# Acute hypoxemia due to right-to-left shunt via a patent foramen ovale during left internal thoracic artery to left anterior descending artery anastomosis in off-pump coronary artery bypass grafting: a case report

**DOI:** 10.1186/s40981-023-00607-x

**Published:** 2023-03-16

**Authors:** Yuki Okutomi, Takeyuki Sajima, Atsushi Yasuda, Shigehito Sawamura

**Affiliations:** Department of Anesthesia and Critical Care, Teikyo University School of Medicine, 2-11-1 Kaga, Itabashi, Tokyo, 173-8606 Japan

**Keywords:** Right-to-left shunt, Patent foramen ovale, Off-pump coronary artery bypass grafting, Hypoxemia, Aortic root enlargement, Atrial septal aneurysm

## Abstract

**Background:**

A right-to-left shunt via a patent foramen ovale (PFO) during off-pump coronary artery bypass (OPCAB) may result in difficulties in oxygenation and circulatory management. We herein present a case of a marked shunt via a PFO during OPCAB.

**Case presentation:**

A 74-year-old man who had aortic root enlargement, compressing the right atrium, and an atrial septal aneurysm, underwent OPCAB. When the heart was fixed for the anastomosis of the left anterior descending artery, sudden hypoxemia and hypotension were observed. Intraoperative transesophageal echocardiography (TEE) showed a right-to-left shunt via a PFO that was unnoticed preoperatively. After the anastomosis was completed, TEE revealed no shunt through the PFO.

**Conclusions:**

We should check for a PFO in case of an atrial septal aneurysm. Compression of the right atrium is considered an important anatomical risk of the right-to-left shunt in OPCAB.

## Background

The right-to-left shunt that occurs during off-pump coronary artery bypass (OPCAB) is purportedly caused by increasing right atrial pressure or pulmonary artery pressure while lifting and stabilizing the heart to expose the target coronary artery [1]. These heart positionings are especially needed during right coronary artery (RCA) or left circumflex anastomosis because the target coronary artery is located behind the heart. Conversely, there are few cases of right-to-left shunt occurrence during left anterior descending artery (LAD) anastomosis in OPCAB. We report a case of acute hypoxemia caused by a right-to-left shunt via a patent foramen ovale (PFO) during left internal thoracic artery to LAD anastomosis in OPCAB in a patient with an atrial septal aneurysm and an enlarged aortic root.

## Case presentation

**Fig. 1:**
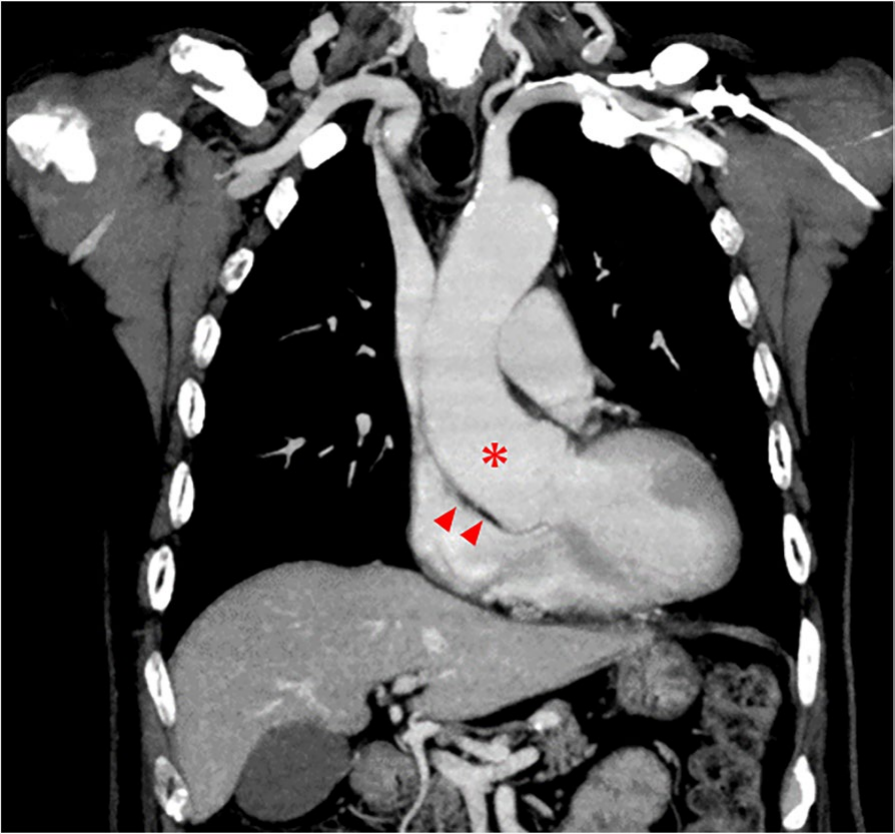
Coronal computed tomography section of the heart. A remarkably enlarged aortic root (asterisk) compressed the right atrium (arrowhead)

A 74-year-old man was transported to our hospital due to fever. His medical history included epilepsy, smoking 20 cigarettes per day for 50 years, and medications such as sodium valproate, clobazam, and phenobarbital. Computed tomography (CT) revealed a compression fracture of the third lumbar vertebra, an abdominal aortic aneurysm, and coronary artery stenosis. Aortic root enlargement was shown on the CT (Fig. 1). The patient was scheduled to undergo OPCAB.

Preoperative transthoracic echocardiography (TTE) showed an ejection fraction of 48% and normal wall motion. The presence of a PFO was not checked. He had mild aortic valve regurgitation with a Valsalva diameter of 51 mm, sinotubular junction of 48 mm, ascending aortic diameter of 43 mm, descending aortic diameter of 37 mm, and abdominal aortic diameter of 58 mm. Coronary angiography revealed stenoses of 90% in the posterior descending branch, 75% in the left main trunk, and 90% in the mid portion of the LAD.

**Fig. 2:**
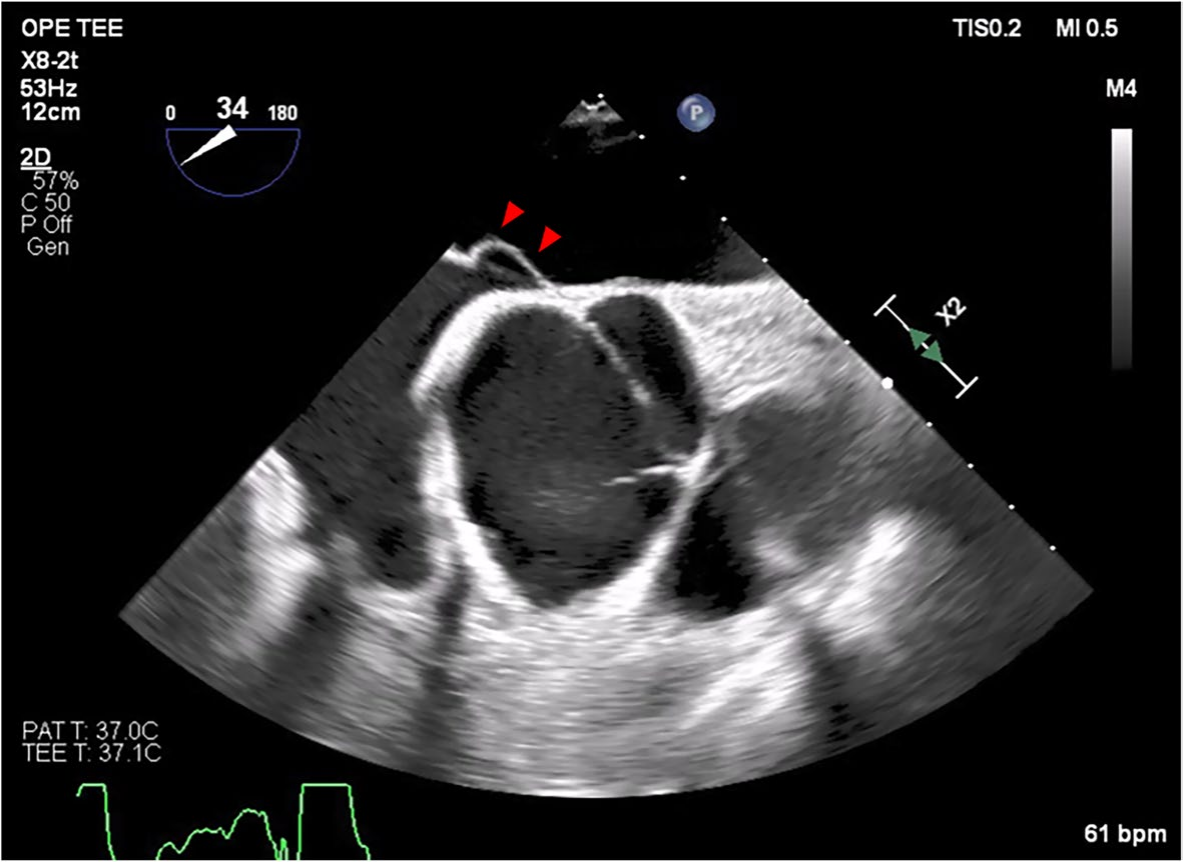
Intraoperative transesophageal echocardiography: aortic valve short axis view. An atrial septal aneurysm with a 10-mm deviation of the atrial septal tissue into the left atrium (arrowhead)

In the operation room, an arterial catheter (FloTrac, Edwards Lifesciences, Irvine, CA, USA) was inserted, followed by induction of anesthesia with midazolam 3 mg, fentanyl 350 µg, and rocuronium 80 mg and maintenance with remifentanil 0.15 µg/kg/min and sevoflurane 0.6–0.8 minimum alveolar concentration. A central venous catheter (PreSep, Edwards Lifesciences, Irvine) was inserted in the right internal jugular vein. Standard monitors, transesophageal echocardiography (TEE), bispectral index monitoring (Covidien, Dublin, Ireland), and cerebral regional oxygen saturation monitoring (INVOS 5100C, Medtronic, Dublin, Ireland) were used. Intraoperative TEE showed aortic root enlargement, compression of the right atrium, and an atrial septal aneurysm, which deviated the atrial septal tissue 10 mm from the atrial plane into the left atrium, but we did not search for the presence of a PFO at that time (Fig. 2).

Using the Octopus Tissue Stabilizer® (Medtronic), anastomosis was performed in the order of saphenous vein graft to the posterior descending artery, right internal thoracic artery to the high lateral branch, and saphenous vein graft to diagonal branch (D1) of the left coronary artery. Atrial fibrillation occurred during the anastomosis of the saphenous vein graft to D1 of the left coronary artery. After two cardioversions, the electrocardiogram returned to sinus rhythm. Then, anastomosis of the RCA and left circumflex artery was performed.

**Fig. 3:**
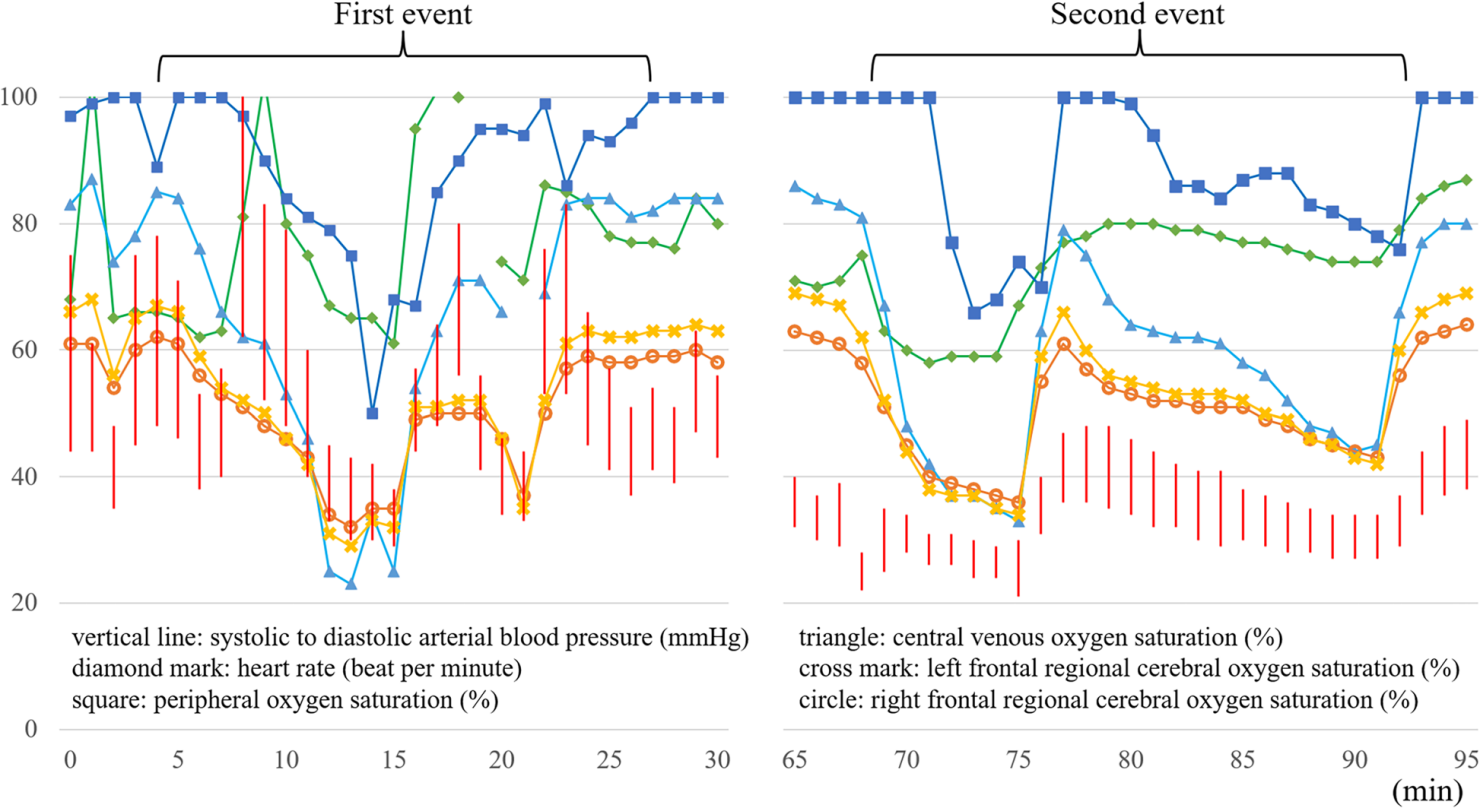
Hemodynamic trends at the events. Vital trends at the time of the event with desaturation and hypotension. Five minutes before the first central venous oxygen saturation drop as zero minutes. The marks in this figure are as follows: vertical line: systolic to diastolic arterial blood pressure (mmHg), diamond mark: heart rate (beat per minute), square: peripheral oxygen saturation (%), triangle: central venous oxygen saturation (%), cross mark: left frontal regional cerebral oxygen saturation (%), circle: right frontal regional cerebral oxygen saturation (%)

For the left internal thoracic artery to LAD anastomosis, the area near the right ventricular outflow tract (RVOT) was fixed using the Octopus Tissue Stabilizer®, resulting in a sudden decrease in central venous oxygen saturation (ScvO_2_) from 84 to 23% and regional cerebral oxygen saturation from 67% in the right frontal region/62% in the left frontal region to 29%/32% over 8 min. This was followed by a decrease in the peripheral oxygen saturation (SpO_2_) from 100 to 49%. Converse to the decrease in oxygenation, the cardiac index increased from 2.0 to 3.3 l/min/m^2^ and then decreased to 1.8 l/min/m^2^ with a decrease in systolic blood pressure from 110 to 38 mmHg) (Fig. 3).

**Fig. 4:**
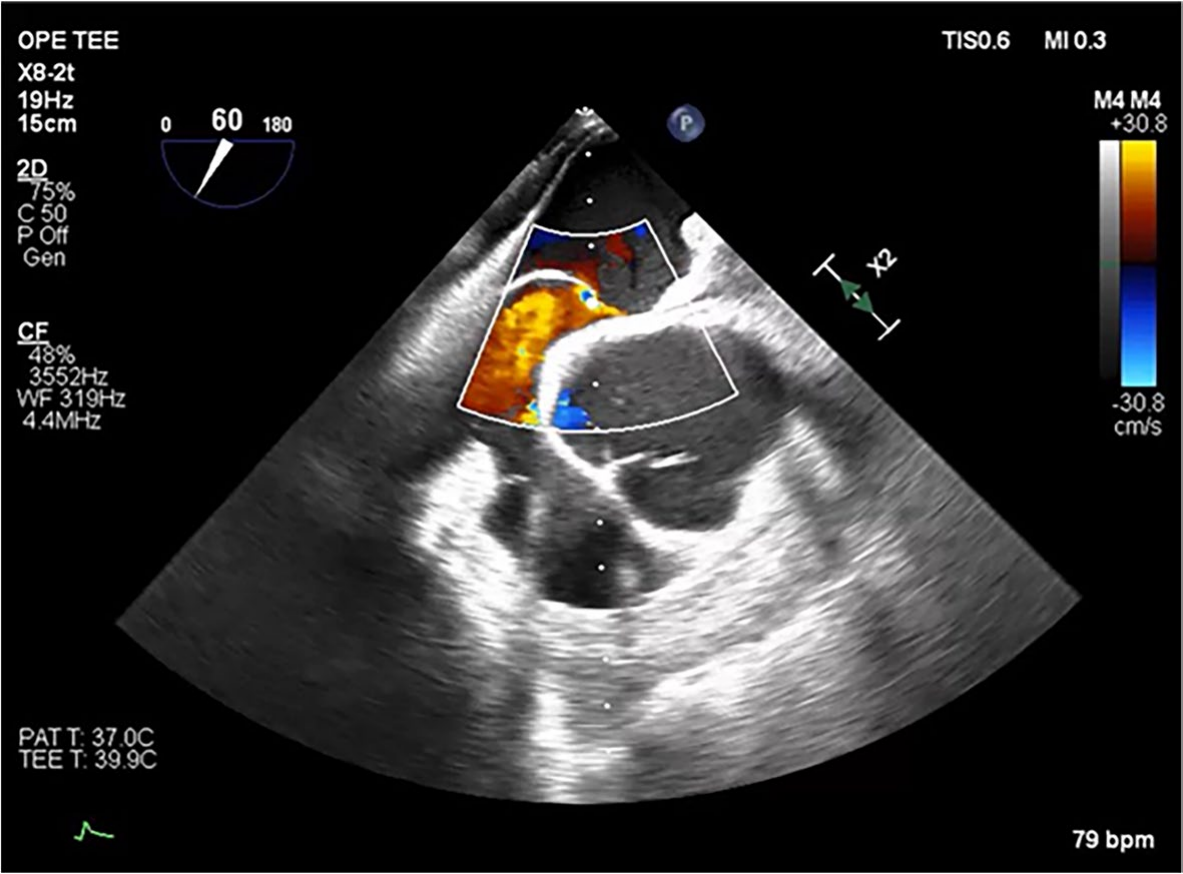
Doppler image of aortic valve short axis view on transesophageal echocardiography. A marked right-to-left shunt through the patent foramen ovale

With these declines, we performed a recruitment maneuver because the TEE showed atelectasis, and fluid resuscitation was performed for possible hypovolemia. However, these methods failed to improve his condition. When the Octopus Tissue Stabilizer® that immobilized the area near the RVOT was released, his blood pressure gradually improved. ScvO_2_, regional cerebral oxygen saturation, and SpO_2_ also returned to their pre-fixation values over 8 min. Using the Octopus Tissue Stabilizer® to immobilize the heart again, ScvO_2_, regional cerebral oxygen saturation, SpO_2_, and blood pressure decreased again. The patient’s central venous pressure (CVP) increased from 5 to 15 mmHg. The TEE revealed a PFO that had been unnoticed preoperatively and a marked right-to-left shunt (Fig. 4).

We hypothesized that the increased afterload in the right ventricular system resulted in a right-to-left shunt from the PFO. After the Octopus Tissue Stabilizer® was released again and the patient’s condition improved, we discontinued positive end-expiratory pressure to reduce pulmonary vascular resistance and minimized fluid administration to decrease right atrial pressure. Then, cardiac immobilization for left internal thoracic artery to LAD anastomosis was tried again while the ScvO_2_ and regional cerebral oxygen saturation were closely monitored. After the anastomosis was completed, TEE revealed no shunt through the PFO.

The operative time was 431 min, anesthesia time was 498 min, blood loss was 5838 mL, total administration of intravenous crystalloid and colloid solutions was 8700 mL, red blood cell transfusion was 2240 mL, and fresh frozen plasma transfusion was 1200 mL. The TTE performed 9 days after the operation showed no shunt via the PFO.

## Discussion

In this case, a rapid decrease in SpO_2_ and blood pressure occurred during left internal thoracic artery to left anterior descending artery anastomosis, and the TEE revealed a right-to-left shunt through a previously undetected PFO. Fixing the area near the RVOT using the Octopus Tissue Stabilizer® and distorting the heart due to enlargement of the aortic root probably contributed to this.

OPCAB involves the lifting, rotating, and compression of the heart with stabilizers to expand the field of view during anastomosis. RVOT stenosis and mitral regurgitation are associated with hypotension, which often necessitates fluid resuscitation, administration of vasoactive agents, and adjustment of the position of the heart.

PFO is present in 20–25% of adults; atrial septal aneurysm is associated with the presence of PFO as well as an increased size of PFO [2–4]. Notably, PFO is a valve-like structure that opens and closes with increased right atrial pressure, resulting in a transient or permanent right-to-left shunt. Pulmonary hypertension and pulmonary artery thromboembolism are the most common conditions that cause increased right atrial pressure, and RVOT stenosis due to OPCAB also increases right atrial pressure. There were reports of the identification of new right-to-left shunting in 2 of 11 patients with preoperatively noted PFO [5] and reports of desaturation due to a right-to-left shunt via the PFO [1, 6]. In this case, intraoperative TEE showed an atrial septal aneurysm and that the patient had a PFO. Furthermore, CVP increased during LAD anastomosis, which suggests a rapid increase in right atrial pressure. The fixation of the area near the RVOT at the time of LAD anastomosis, which probably resulted in outflow tract stenosis, increased right atrial pressure and caused desaturation by a marked right-to-left shunt via the PFO. Subsequently, myocardial ischemia due to desaturation is thought to have caused rapid hypotension.

In addition, anatomical axial deviation of the right atrium, such as tortuosity of the aorta and deformation of the spinal column, facilitates venous return from the inferior vena cava into the left atrium through the foramen ovale [7]. The aortic root enlargement and compression of the right atrium by the aortic root likely contributed to the prominent right-to-left shunt via the PFO in our case.

In general, stenosis of the RVOT causes hypotension by decreasing the preload of the left ventricular system. In this case, hypotension appeared late, possibly due to the marked right-to-left shunt via the PFO having compensated for the reduced preload of the left ventricular system due to RVOT stenosis. The significant decrease in SpO_2_, ScvO_2_, and regional cerebral oxygen saturation levels was accompanied by a decrease in blood pressure. Because the time between the drop in ScvO_2_ level and the drop in blood pressure was short, ranging from a few seconds to a few minutes, a quick response was required. Although we considered conversion to an on-pump procedure, we did not convert because the shunt disappeared after cessation of lifting, rotation, and compression of the heart.

As shown in this case, if an atrial septal aneurysm is present, we should suspect an existing PFO. We should check for the presence of a PFO, especially when patients have an anatomical axial deviation of the right atrium and when operations that cause right atrial pressure elevation are performed.

In conclusion, we report a case of OPCAB, in which the fixation of the area near the RVOT caused a marked right-to-left shunt via a PFO that had not been identified preoperatively. A PFO should be checked for in case of an atrial septal aneurysm. Aortic root enlargement and compression of the right atrium are considered important anatomical causes of the right-to-left shunt via a PFO.

## Data Availability

Not applicable.

## Abbreviations

CT: Computed tomography
CVP: Central venous pressure
LAD: Left anterior descending artery
OPCAB: Off-pump coronary artery bypass
PFO: Patent foramen ovale
RCA: Right coronary artery
RVOT: Right ventricular outflow tract
ScvO_2_: Central venous oxygen saturation
SpO_2_: Peripheral oxygen saturation
TEE: Transesophageal echocardiography
TTE: Transthoracic echocardiography
